# Peripheral blood mononuclear cell number and paracrine function in responses to a 50‐km trail race: An exploratory study

**DOI:** 10.14814/phy2.70255

**Published:** 2025-02-19

**Authors:** Rian Q. Landers‐Ramos, Katherine Kim, James Heilman, William S. Evans, Odessa Addison, Sushant M. Ranadive, Steven J. Prior

**Affiliations:** ^1^ Department of Kinesiology Towson University Towson Maryland USA; ^2^ Department of Kinesiology University of Maryland College Park Maryland USA; ^3^ Department of Exercise Science Elon University Elon North Carolina USA; ^4^ Department of Physical Therapy and Rehabilitation Science University of Maryland Baltimore Maryland USA; ^5^ Department of Veterans Affairs Baltimore Veterans Affairs Medical Center Geriatric Research, Education and Clinical Center Baltimore Maryland USA

**Keywords:** paracrine function, peripheral blood mononuclear cell, proliferation, prolonged exercise

## Abstract

Peripheral blood mononuclear cells (PBMCs) represent a heterogeneous mix of cells with paracrine functions that may be altered following prolonged exercise. We determined the effect of ultramarathon running on PBMC paracrine function and PBMC subtype number. Recreational athletes participated in a 50 km ultramarathon. Blood was sampled from *N* = 7 at baseline, 10 km, 50 km, and 24 h post‐race. PBMCs were isolated and cultured, and conditioned media was used for a HUVEC‐based proliferation assay. CD31+, CD3+, and CD31+/CD3+ PBMCs were quantified at each time point. Proliferation increased from baseline to 50 km (*p* = 0.004) and was reduced from 50 km to 24 h post (*p* = 0.008). There was an increase in CD31+ PBMCs after 50 km (*p* = 0.014), returning to baseline at 24 h post‐race (*p* = 0.246). CD3+ PBMC and CD31+/CD3+ PBMC numbers were reduced after 50 km (*p* = 0.001 and *p* = 0.002, respectively), returning to baseline levels 24 h post‐race (*p* = 0.190 and *p* = 0.315, respectively). PBMC paracrine activity following a 50 km enhances endothelial cell proliferation. Alterations in PBMC subtypes after 50 km suggest a protective role of PBMCs in response to prolonged stresses of ultramarathon running.

## 1 INTRODUCTION

Peripheral blood mononuclear cells (PBMCs) represent a heterogeneous mix of cells, many of which exhibit paracrine functions, promote angiogenic potential in surrounding tissues, and are associated with endothelial cell function (Hill et al., 2003; Hoetzenecker et al., 2015; Matsui et al., 2003). Preconditioning of PBMCs with growth factors (Avraham‐Davidi et al., 2013) or oxygen/glucose deprivation (Hatakeyama et al., 2019) has been utilized to enhance the angiogenic properties of these cells using in vitro experiments, and we have previously found that the angiogenic paracrine activity of PBMC subtypes is enhanced in regular exercisers (Landers‐Ramos et al., 2015). This suggests that exercise may alter or “condition” the secretome of PBMCs in vivo to impact angiogenic potential or support nearby endothelial cell function.

CD31+ cells represent approximately 30%–35% of total PBMCs (Kim et al., 2011) and exhibit strong vasculogenic potential through the induction of angiogenic gene expression (Kim et al., 2010, 2011; Landers‐Ramos et al., 2017). Surface markers present along with CD31, particularly CD3, may lend to a cell population with stronger vasculogenic properties that support endothelial cell health (Hur et al., 2007; Kushner, Weil, et al., 2010; Kushner, MacEneaney, et al., 2010). Like progenitor cells, CD31+/CD3+ PBMCs are mobilized in response to acute coronary events (Weil et al., 2011) and acute exercise and typically egress into their target tissue, returning to resting levels after several hours (Möbius‐Winkler et al., 2009; Ross et al., 2024). Physical exercise induces acute hypoxia in muscle tissue that promotes PBMC migration to muscle (Adams et al., 2004) where PBMCs exert paracrine effects (Adams et al., 2008; Bonsignore et al., 2010; Landers‐Ramos et al., 2015). Thus, the vasculogenic potential of PBMCs is dependent on (a) their mobilization into circulation, increasing the likelihood of transport to ischemic areas, and (b) the paracrine mechanisms to promote endothelial cell function in target tissues.

Participation in ultra‐endurance events among recreational athletes has risen substantially in recent years (Scheer, 2019). While endurance exercise meeting the ACSM recommendations (Garber et al., 2011) improves vascular function (Dawson et al., 2013; Liu et al., 2022), prolonged endurance exercise, such as ultramarathon running, may lead to unaccustomed strain on the vasculature (Adams et al., 2008; Bonsignore et al., 2002, 2010; Landers‐Ramos et al., 2021). Conversely, we have previously shown that brachial artery endothelial function is maintained following an ultramarathon (Ranadive et al., 2024), and others (Goussetis et al., 2009) have suggested that altered PBMC number and function in response to prolonged exercise, although short‐lived, may be involved in preventing vascular damage. The current study aimed to determine the effect of ultramarathon running on PBMC paracrine function and PBMC subtype number in healthy endurance‐trained adults. We hypothesized that (1) conditioned media (CM) generated from PBMCs collected after an ultramarathon would increase endothelial cell proliferation, with the effects of the CM returning to baseline 24 h post‐race, and (2) an ultramarathon would increase PBMC subtype numbers, with values returning to baseline levels 24 h post‐race.

## 2 MATERIALS AND METHODS

### 2.1 Ethical approval

The University of Maryland College Park Institutional Review Board approved all study procedures (#1300927). Written informed consent was obtained for all participants, and all study procedures adhered to those outlined in the Declaration of Helsinki.

### 2.2 Participants and fitness status

Eleven healthy recreational runners between 30 and 60 years of age and registered for a local 50 km race were recruited for this study. Exclusion criteria included a body mass index (BMI) outside the range of 17–35 kg/m^2^, the presence of acute illness, current smoker status, or a history of cardiovascular disease. After screening for eligibility, maximal oxygen consumption (VO_2_ max) was quantified via indirect calorimetry (COSMED, Rome, Italy) as described previously (Landers‐Ramos et al., 2022).

### 2.3 Procedures and blood sampling

A total of 30 mL of blood was drawn on race day (before race start, 10 km into the race, and within 60 min (±10 min) after crossing the finish line of the 50 km) using sterile ethylenediaminetetraacetic acid (EDTA) vacutainer tubes (BD Biosciences #366643, San Jose, CA). On the day following the race, 10 mL of blood was collected during a visit scheduled approximately 24 h after each participant's finishing time. The first lap (10 km) was chosen as a comparison distance due to it being considered a “standard” rather than “prolonged” bout of aerobic exercise.

### 2.4 PBMC isolation

PBMCs were isolated from blood samples using density gradient centrifugation (Ficoll; GE Healthcare #17–1440, Pittsburgh, PA). Cells were counted following isolation with a Bio‐Rad™ TC20™ automated cell counter to determine the density of cells in suspension for both flow cytometry and generation of CM.

### 2.5 Generation and collection of conditioned media

A total of 3 × 10^6^ isolated PBMCs were incubated at 37 C and 5% CO_2_ in a 6‐well cell culture plate (NEST #703003, Wuxi, China) for 24 h in 3 mL/well of Endothelial Cell Growth Basal Medium‐2 (EBM™‐2, Lonza #00190860, Basel, Switzerland) supplemented with 1X Penicillin‐Streptomycin (Landers‐Ramos et al., 2017). After 24 h, CM was harvested and aliquoted for storage at −80 C for future assays.

### 2.6 Cell proliferation assay

Human umbilical vein endothelial cells (HUVECs, Lonza #C2519A, Basel, Switzerland) were seeded into a 96‐well tissue culture plate (Greiner Bio‐One #655090, Monroe, North Carolina) at a density of 30,000 cells/mL (Landers‐Ramos et al., 2017). After the cells were given 8 h to attach to the bottom of each well and approach 50% confluency, CM from each time point for each subject was added to the wells to be analysed in duplicate. After a 24‐h incubation period, a fluorometric cell proliferation assay kit (BioVision #K307–1000, Milipitas, CA) was used to assess proliferation, following the manufacturer instructions. The plate was then gently rocked for 15 min at room temperature while being protected from light. The fluorescence of the cells in each well was then measured using a microtiter plate reader (Agilent BioTek FLx800TBIE, Santa Clara, CA) at Ex/Em = 480/538 nm. HUVEC proliferation was measured as relative fluorescence units (RFU) from each well of cells treated with PBMC‐CM from each time point.

### 2.7 Flow cytometry

A separate aliquot of isolated PBMCs was stained for flow cytometry. After an Fc receptor blocker (Miltenyi Biotech #130–059‐901, San Diego, CA) was added to cell samples, PBMCs were incubated with APC‐CD3 (R&D Systems Cat# FAB100, RRID:AB_356978), V450‐CD31 (BD Biosciences Cat# 561653, RRID:AB_10896326), and separate fluorescence minus one (FMO) controls were used to enhance the accuracy of cell marker identification. Single‐stained and unstained controls were also prepared for fluorescence‐activated cell sorting calibration. Each control vial and experimental sample containing both cells and antibody was prepared with a density of 2 × 10^6^ cells/mL within 5 mL round‐bottom polystyrene tubes (Fisher Scientific #14–956‐3D, Hampton, NH). CD3+, CD31+, and CD3+/CD31+ cell populations were then identified via the FACSCanto II system (BD Biosciences, San Jose, CA) per 100,000 events. Flow cytometry data were analyzed using FCS Express software 6 (De Novo Software, Glendale, CA).

### 2.8 Statistical analysis

Data are reported as means ± SD. One‐way repeated measures ANOVAs (SPSS version 22, IBM, Armonk, NY) were used to analyze cell proliferation and flow cytometry data. When a significant main effect of time was noted, post hoc analyses were performed using Tukey's LSD. When significant, BMI and VO_2max_ were included in the model as covariates. The criterion for statistical significance was *p* ≤ 0.05.

## 3 RESULTS

### 3.1 Participants

Four of the eleven initial subjects were excluded from analyses due to missing race‐day blood samples. One additional participant was excluded from flow cytometry analyses due to technical issues. Participant characteristics for the remaining subjects can be found in Table 1. The average time to complete the 50 km race was 6:45.00 ± 0:44.4.

**TABLE 1:** Subject characteristics.

Sex (M/F)	6/1
Age (y)	40 ± 8
BMI (kg/m^2^)	25 ± 3
VO_2max_ (mL/kg/min)	48.9 ± 3.8
Resting SBP (mmHg)	133 ± 6
Resting DBP (mmHg)	80 ± 7
Resting MAP (mmHg)	95 ± 6
Resting HR (bpm)	61 ± 10

*Note*: Data are means ± SD. Subject characteristics from a larger cohort including these participants has been previously published (Landers‐Ramos et al., 2022; Ranadive et al., 2024).

Abbreviations: BMI, body mass index; DBP, diastolic blood pressure; HR, heart rate; MAP, mean arterial pressure; SBP, systolic blood pressure; VO_2max_, maximal oxygen consumption.

### 3.2 PBMC effects on endothelial cell proliferation

**FIGURE 1:**
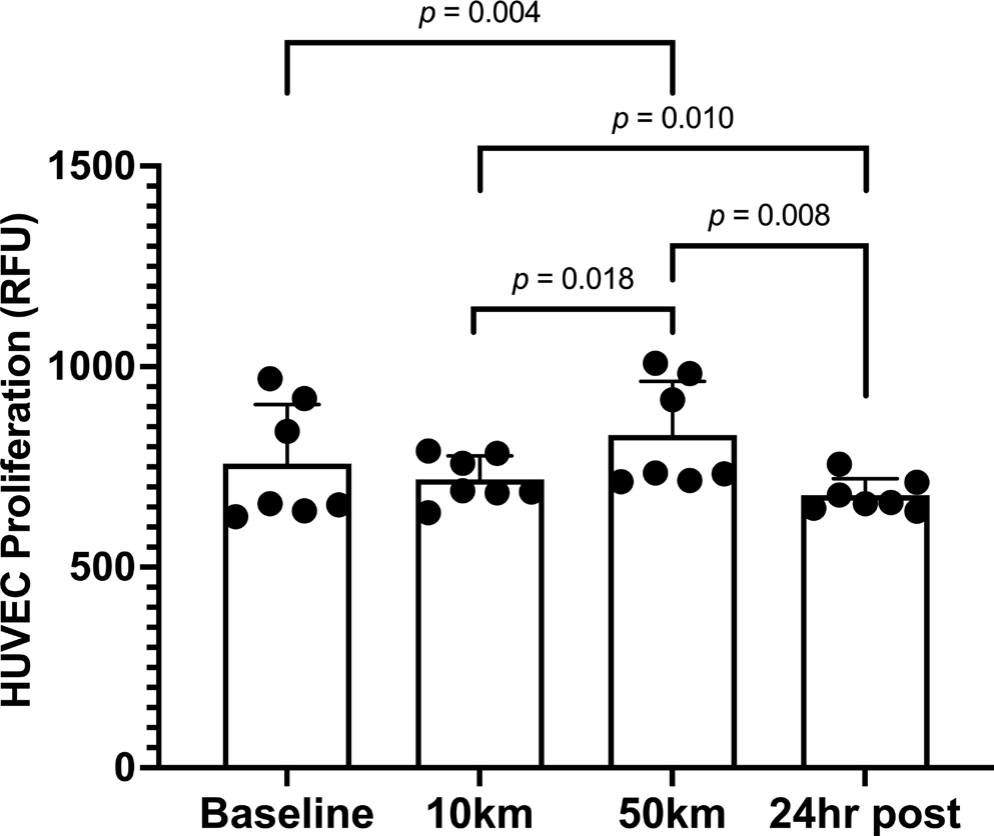
HUVEC proliferation in response to PBMC conditioned medium at baseline, 10 km, 50 km, and 24 h post‐race. Statistical significance was accepted at *p* ≤ 0.05. Data are reported as means ± SD.

There was a significant effect of the ultramarathon on PBMC CM‐induced endothelial proliferation (*p* = 0.017; Figure 1). Proliferation did not change between baseline and 10 km (*p* = 0.332), increased significantly from baseline to 50 km (*p* = 0.004), and was significantly reduced from 50 km to 24‐h post (*p* = 0.008) such that values returned to baseline levels at 24‐h post (*p* = 0.090). Proliferation was significantly elevated between 10 km and 50 km (*p* = 0.018) and significantly reduced from 10 km to 24‐h post‐race (*p* = 0.010).

### 3.3 PBMC subtype numbers

**FIGURE 2:**
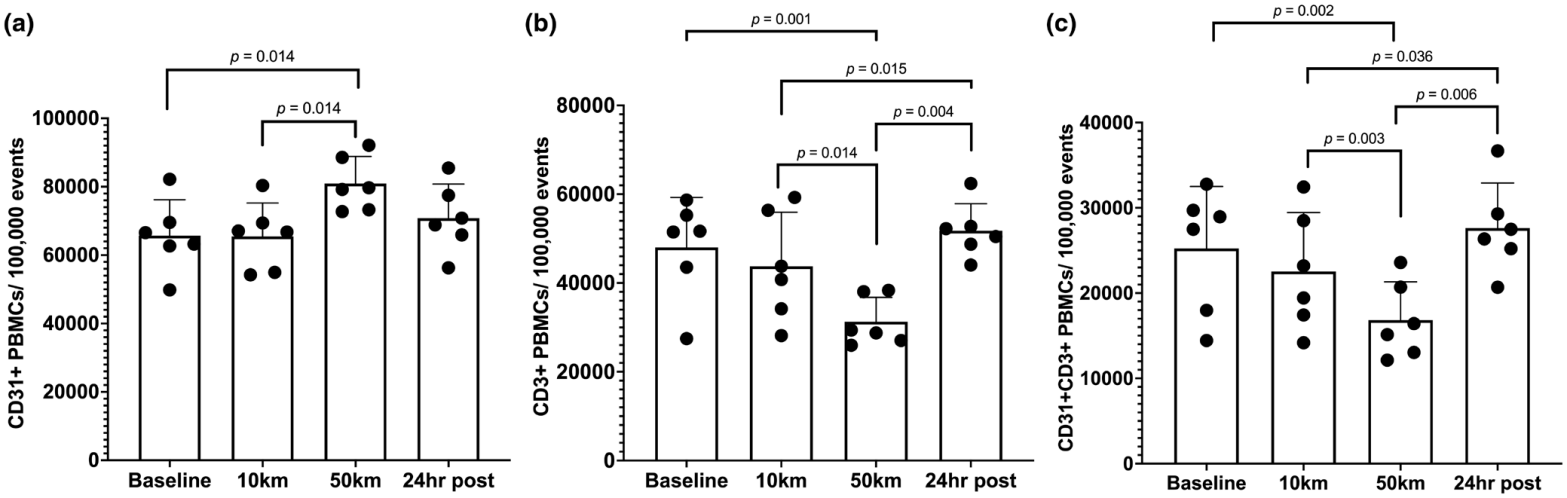
Quantification of (a) CD31+ PBMCs, (b) CD3+ PBMCs, and (c) CD31+/CD3+ PBMCs at baseline, 10 km, 50 km, and 24 h post‐race. Statistical significance was accepted at *p* ≤ 0.05. Data are reported in means ± SD.

There was a significant main effect of the ultramarathon on CD31+ PBMC number (*p* = 0.005; Figure 2a). There was no change in CD31+ PBMC number at 10 km (*p* = 0.913) but there was a significant increase in CD31+ PBMCs after 50 km (*p* = 0.014 vs. baseline and *p* = 0.014 vs. 10 km). CD31+ PBMC number appeared to decrease at 24 h post‐race but was not different from baseline (*p* = 0.246) or 50 km (*p* = 0.126).

The main effect of the ultramarathon on CD3+ PBMCs (*p* = 0.008; Figure 2b) was significant. CD3+ PBMC number was unchanged at 10 km (*p* = 0.220) but went down significantly after 50 km (*p* = 0.001 vs. baseline, *p* = 0.014 vs. 10 km). 24 h post‐race, CD3+ PBMC number recovered significantly from 50 km (*p* = 0.004) and was similar to baseline levels.

There was a main effect of the ultramarathon on CD31+/CD3+ PBMCs (*p* = 0.022; Figure 2c). The 10 km distance did not impact CD31+/CD3+ PBMC number (*p* = 0.164), but there was a significant decline in CD31+CD3+ PBMC number at the 50 km (*p* = 0.002 vs. baseline and *p* = 0.003 vs. 10 km). CD31+/CD3+ PBMC number recovered at 24 h post‐race (*p* = 0.006 vs. 50 km and *p* = 0.315 vs. baseline).

## 4 DISCUSSION

In this study, we present evidence that PMBC paracrine activity following a 50 km run enhances endothelial cell proliferation. We also found that PBMC subtypes respond differently to prolonged running, with CD31+ PBMCs being elevated after 50 km and CD3+ and CD31+/CD3+ PBMCs being reduced in response to the 50 km run. Both PBMC numbers and PBMC paracrine‐related proliferation returned to baseline 24 h post race. Further, the 10 km distance did not elicit any significant responses.

We found that CD31+ PBMCs were significantly elevated after the 50 km race. More than 90% of hematopoietic stem cells, progenitor cells, and monocyte progenitor cells express CD31 (Kim et al., 2011). CD31 is constitutively expressed on the surface of circulating monocytes, which function predominantly through paracrine mechanisms (Rehman et al., 2003, 2004). The timeframe of elevated CD31+ PBMCs post‐50 km coincides with the increased proliferative capacity of PMBC‐CM on endothelial cells, suggesting a link between these outcomes. For instance, CD31+ PBMCs mobilized at the same time point would enrich the overall population of PBMCs and potentially contribute to the observed endothelial proliferation through paracrine mechanisms after 50 km. CD31 is also expressed on mature endothelial cells (Hwang et al., 2014); thus, an alternative explanation for the increased CD31+ PBMC number may involve denudation from the endothelium or other tissues in response to the physiological stresses of prolonged exercise (Erdburegger et al., 2010). While we have previously shown an exercise duration of ~30–60 min does not contribute to endothelial cell denudation (Sapp et al., 2019), the prolonged nature of an ultramarathon may result in differential effects.

Some studies have previously found that CD31+/CD3+ PBMCs increase with acute exercise (Ross et al., 2016, 2018; Santosa et al., 2016), while others report no mobilization response (Lansford et al., 2016; Niemiro et al., 2017; Shill et al., 2016). However, no other studies that we are aware of have demonstrated a reduction in CD31+/CD3+ or CD3+ PBMCs with exercise. One explanation for these discrepancies may be differences in the timeframe of blood sampling. For example, studies reporting increases in CD31+/CD3+ PBMC number sampled blood immediately following exercise, with at least one study demonstrating CD31+/CD3+ levels returning to baseline within 1 h post‐exercise (Ross et al., 2016, 2018; Shill et al., 2016). In our study, blood samples were taken approximately 1 hr post‐race. The time course of CD31+/CD3+ and CD3+ PBMC mobilization may involve a peak during and/or immediately following exercise, with cell egress from circulation into target tissue soon thereafter to facilitate repair/remodeling of the endothelium. In support of this concept, we have reported that flow‐mediated dilation was maintained after the 50 km run in these same individuals (Ranadive et al., 2024) suggesting that some mechanisms are in place to preserve endothelial function in response to prolonged endurance exercise. Future studies are needed to experimentally establish whether this time course exists and whether PBMC uptake reflects improved or maintained endothelial function.

We did not see differences in any outcome at the 10 km time point. Our findings are consistent with those of others following moderate‐intensity exercise of a timeframe similar to 10 km completion (Lansford et al., 2016; Niemiro et al., 2017). The relative intensity of the initial 10 km may not have been high enough to elicit a significant response from our participants (O'Carroll et al., 2019; Ross et al., 2016; Shill et al., 2016). In the absence of high‐intensity exercise, the duration of the 50 km (6:45.00 ± 0:44.4) appears sufficient to elicit changes in PBMC subtype number. Goussetis et al. found that endothelial progenitor cells increased 11‐fold following a 246 km ultramarathon, and this reflected changes in inflammation (Goussetis et al., 2009). In these participants, we previously reported that IL‐6 and calprotectin are increased after 50 km (Landers‐Ramos et al., 2022), potentially serving as a stimulus for CD31+ PBMC mobilization or uptake of CD3+ and CD31+/CD3+ PBMCs. These inflammatory factors returned to baseline levels 24 h post‐exercise, reflecting the timeframe for our PBMC outcomes.

This study was performed in a race venue, which limited the number of participants that could be tested due to race day logistics. The results present promising evidence for prolonged running serving as a potent conditioning stimulus for circulating PBMCs, resulting in enhanced paracrine‐related proliferation capacity. We have previously shown that the secretome from endurance athletes versus inactive individuals can have a significant impact on HUVEC capillary‐like network formation due largely to the release of S100A8 and S100A9 (Landers‐Ramos et al., 2015). Unfortunately, we were limited by the number of CM aliquots to perform additional functional assays or to mechanistically assess specific factors present in the secretome that are responsible for enhancing endothelial proliferation. Further, in this study, we used a fixed number of PBMCs to create the CM for our HUVEC proliferation assay. This standardization helps to isolate the paracrine function from the changes in PBMC numbers. However, as PBMC numbers can increase with exercise (Bonsignore et al., 2010; Lutz et al., 2016; Ross et al., 2016), the additive influence of paracrine actions and higher cell numbers would likely magnify the physiological impact. Future studies may wish to explore the effects of different concentrations of PBMCs to reflect exercise‐induced changes in PBMC numbers. While CD31+ cells and CD3+ cells comprise up to 35% and 60% of PBMCs, respectively (Kim et al., 2011; Kleiveland, 2015), our findings do not allow us to state that any subpopulation of PBMCs is solely responsible for endothelial proliferation. Finally, PBMCs regulate vascular repair in a multifuctional manner, which, in addition to proliferation, includes endothelial cell adhesion and engraftment (Hur et al., 2004; Kim et al., 2010; Kushner, MacEneaney, et al., 2010), expression and production of nitric oxide (Hur et al., 2004; Jenkins, Landers, Prior, et al., 2011; Jenkins, Landers, Thakkar, et al., 2011), and other paracrine‐mediated effects such as capillary‐like tube formation (Hur et al., 2004; Landers‐Ramos et al., 2015) and anti‐inflammatory actions on target tissues (Hoetzenecker et al., 2015). Thus, our proliferation findings represent just one component of the complex involvement of PBMCs as regulators of vascular repair.

Though previous studies might suggest that bouts of prolonged endurance exercise may negatively affect vascular endothelium (Bonsignore et al., 2010; Dawson et al., 2008), our results suggest that PBMCs may play a role in preventing vascular damage in response to prolonged stresses of ultramarathon running. However, future studies are needed to further understand the PBMC functions (uptake/integration into existing endothelium vs. paracrine mechanisms) responsible for the preservation of endothelial function during prolonged exercise.

## AUTHOR CONTRIBUTIONS

RQL: conception and design, acquisition and interpretation, drafting and revising the manuscript; KK: acquisition and interpretation, drafting of the manuscript; JH: acquisition and analysis, editing the manuscript; WSE: acquisition, analysis, and interpretation, editing the manuscript; OA: conception and design, acquisition and interpretation, revising; SMR: conception and design, acquisition and interpretation, revising; SJP: conception and design, acquisition and interpretation, revising the manuscript. All authors approved the final version of the manuscript and agree to be accountable for all aspects of the work in ensuring that questions related to the accuracy or integrity of any part of the work are appropriately investigated and resolved. All persons designated as authors qualify for authorship, and all those who qualify for authorship are listed.

## FUNDING INFORMATION

SJP was supported by the Baltimore Veterans Affairs Geriatric Research, Education, and Clinical Center. RQL was supported by the Towson University College of Health Professions.

## CONFLICT OF INTEREST STATEMENT

There are no conflicts of interest to disclose.

## Supporting information


Figure S1.

## Data Availability

The data that support the findings of this study are available from the corresponding author, RQL, upon reasonable request.
