# Supplementary material for: Peripheral blood mononuclear cell number and paracrine function in responses to a 50‐km trail race: An exploratory study
Source: Physiol Rep. 2025 Feb 19;13(4):e70255. doi: 10.14814/phy2.70255 (PMC11839398; doi:10.14814/phy2.70255)
Supplement: Supplementary file 1 — Figure S1. [file PHY2-13-e70255-s001.zip › PHYSREP-2024-12-832-f01-z-.docx]

**Supplemental Figure Captions**

**Supplemental Figure 1.** Selected Unstained vs. stained FACS analysis and gating strategy. A) CD3+ population gated out from monocyte population based on APC-A fluorescence. B) CD3+/CD31+ co-expressing PBMCs gated out from CD3+ population based on V450/50-A fluorescence. C) CD3+/CD31+ PBMC frequency histogram. Range M3 includes all instances of CD3+/CD31+ positive events. Two distinct peaks can be seen; peak M2 corresponds to CD3+/CD31+ PBMCs with lymphocyte lineage; peak M1 corresponds to CD3+/CD31+ PBMCs with monocyte lineage.
