# Supplementary figures and images for: Peripheral blood mononuclear cell number and paracrine function in responses to a 50‐km trail race: An exploratory study
Source: Physiol Rep. 2025 Feb 19;13(4):e70255. doi: 10.14814/phy2.70255 (PMC11839398; doi:10.14814/phy2.70255)

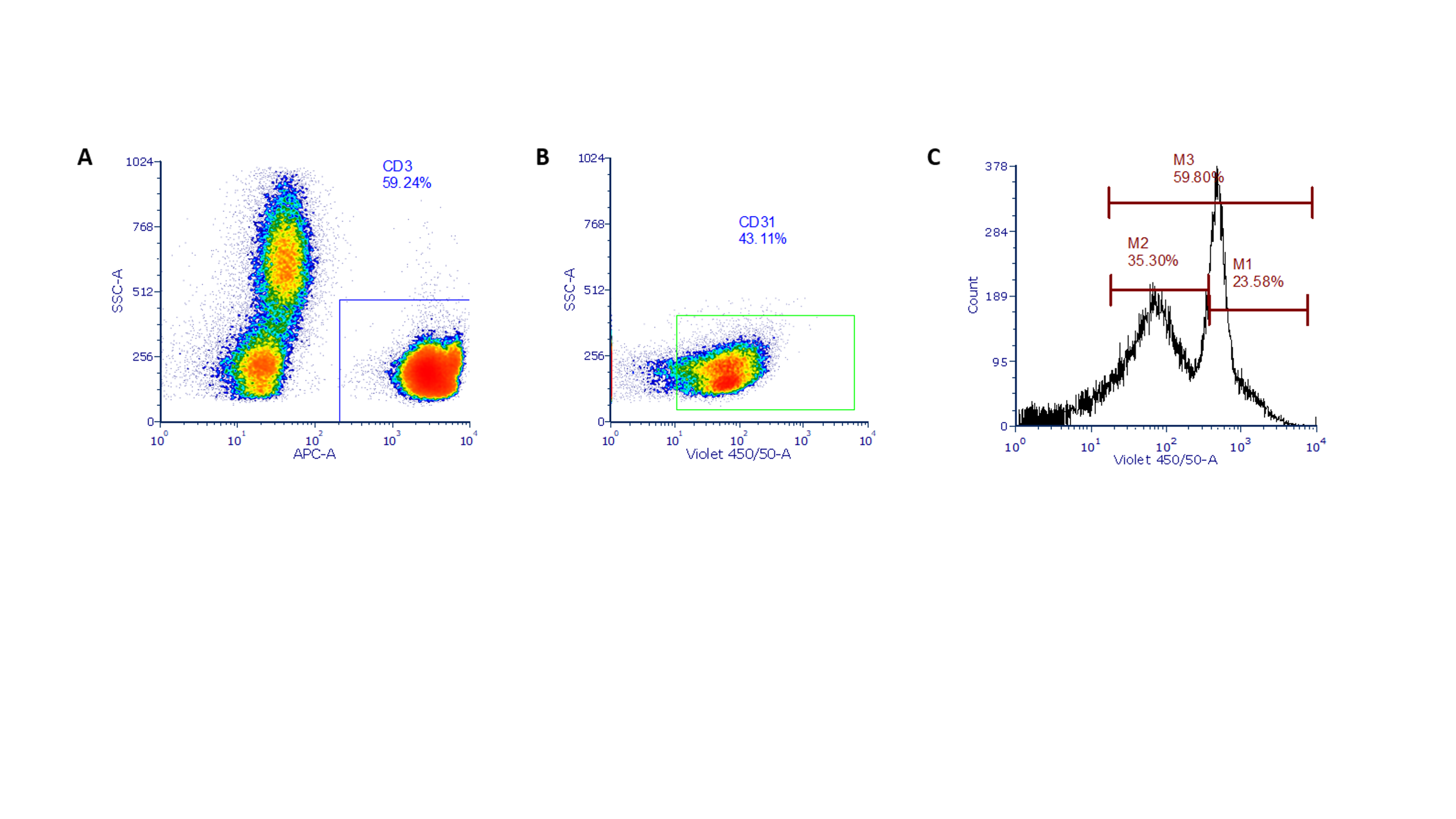

Supplement: Supplementary file 1 — Figure S1. [file PHY2-13-e70255-s001.zip › PHYSREP-2024-12-832-f02-z-.tif]
